# Claudin 18.2 Expression in Gastric Adenocarcinoma: Diagnostic Reproducibility and Clinicopathologic Associations in A Western Cohort

**DOI:** 10.32604/or.2026.075609

**Published:** 2026-05-21

**Authors:** Cristina Díaz del Arco, Luis Ortega Medina, Patricia Barreiro Sanabria, Andrés Sánchez Pernaute, Lourdes Estrada Muñoz, Elena Molina Roldán, María Jesús Fernández Aceñero

**Affiliations:** 1 Department of Legal Medicine, Psychiatry and Pathology, School of Medicine, Complutense University of Madrid, Madrid, Spain; 2 Department of Pathology, Hospital Clínico San Carlos; Health Research Institute of the Hospital Clínico San Carlos (IdISSC), Madrid, Spain; 3 Department of General Surgery, Hospital Clínico San Carlos, Madrid, Spain; 4 Department of Pathology, Rey Juan Carlos Hospital, Móstoles, Madrid, Spain; 5 CNIO Biobank, Spanish National Cancer Research Center (CNIO), Madrid, Spain

**Keywords:** Stomach neoplasms, claudin-18, immunohistochemistry, microsatellite instability, biomarkers, intratumoral, heterogeneity, claudin 18.2 (CLDN18.2)

## Abstract

**Background:** Claudin 18.2 (CLDN18.2) has become a clinically relevant therapeutic target in gastric adenocarcinoma (GC), with zolbetuximab now approved for use in CLDN18.2-positive, HER2-negative advanced disease. The aim of this study was to evaluate the prevalence, intratumoral reproducibility, and clinicopathologic associations of CLDN18.2 expression in a Western cohort of resected GC. **Methods:** CLDN18.2 expression was evaluated by immunohistochemistry in 204 resected GCs arranged in tissue microarrays containing duplicate tumor cores corresponding to the tumor center and invasive front. Correlations between paired cores, clinicopathologic parameters, additional biomarkers (E-cadherin, HER2, p53, mismatch repair (MMR)), and clinical outcomes were analyzed. **Results:** Using the clinical cutoff, 29.9% of cases were CLDN18.2-positive. Inter-core reproducibility was excellent (93.8% concordance, κ = 0.871), indicating minimal intratumoral heterogeneity. CLDN18.2 positivity was consistently associated with male sex, an infiltrative invasive front and absence of necrosis (*p* < 0.05) and occurred more frequently in MMR-proficient tumors (*p* = 0.041 at the 50% cutoff). CLDN18.2-positive cases showed higher rates of strong diffuse membranous E-cadherin staining (*p* < 0.001). At the clinical cutoff, CLDN18.2-positive patients were younger (*p* = 0.049). No associations were found with HER2, p53, recurrence, or cancer-specific survival. However, tumors with extreme expression (Z-score ≥ 250; 14.4%) showed increased Borrmann type IV GC, lymphovascular invasion and cancer-related mortality (*p* = 0.038), with strengthened significance in stage III tumors (disease-specific survival, *p* = 0.026). **Conclusions:** CLDN18.2 shows excellent intratumoral reproducibility and a stable biological profile in GC, supporting its diagnostic reliability in biopsies and value as a predictive biomarker. A subset with extreme expression demonstrated aggressive features, suggesting a potential “claudin-driven” phenotype requiring further study.

## 1 Introduction

Gastric adenocarcinoma (GC) remains a leading cause of cancer mortality worldwide, with limited overall survival despite recent therapeutic advances [1,2]. The identification of clinically actionable biomarkers has therefore become a major focus in GC research.

Claudins are integral components of epithelial tight junctions, where they regulate paracellular permeability, maintain apical–basal polarity, and participate in signaling pathways involved in cell proliferation and differentiation [3,4]. Structurally, they are 20–27 kDa tetraspan transmembrane proteins with two extracellular loops that enable homotypic and heterotypic interactions between adjacent cells [3]. Beyond their barrier function, claudins interact with cytoskeletal and regulatory proteins, influencing cellular architecture and signaling networks. During carcinogenesis, altered claudin expression and mislocalization may disrupt epithelial integrity, facilitate loss of polarity, and contribute to invasion and tumor progression [3,4].

The CLDN18 gene encodes two tissue-specific splice variants: claudin-18.1, predominantly expressed in lung epithelium, and claudin-18.2 (CLDN18.2), physiologically restricted to differentiated gastric epithelial cells [3]. In normal gastric mucosa, CLDN18.2 is confined to tight junction complexes; however, loss of polarity during malignant transformation may increase its membrane accessibility, supporting its development as a therapeutic target [5].

The therapeutic relevance of CLDN18.2 has been established with the development of zolbetuximab, a chimeric IgG1 monoclonal antibody targeting the first extracellular loop of CLDN18.2 and mediating antibody-dependent cellular cytotoxicity and complement-dependent cytotoxicity [3,4]. In the phase III SPOTLIGHT and GLOW trials, the addition of zolbetuximab to first-line chemotherapy significantly improved progression-free and overall survival in patients with CLDN18.2-positive, HER2-negative advanced GC [5,6]. In these pivotal studies, CLDN18.2 positivity was defined as moderate-to-strong membranous staining in ≥75% of tumor cells, establishing a clinical threshold for patient selection.

Despite these advances, important challenges remain. Reported CLDN18.2 prevalence and clinicopathologic associations vary across studies, partly reflecting differences in antibody clones, scoring systems, positivity thresholds, and cohort composition [7,8,9]. In addition, most available data derive from Asian populations, whereas Western cohorts remain comparatively limited. Moreover, although CLDN18.2 testing is increasingly implemented in clinical practice, systematic evaluations of intratumoral heterogeneity and inter-sample reproducibility are scarce, which is particularly relevant for biopsy-based patient selection [10,11,12].

In this context, we examined CLDN18.2 expression in a Western cohort of resected GC, evaluated inter-core concordance as a measure of intratumoral heterogeneity, and investigated associations with clinicopathologic, immunohistochemical, and prognostic features, aiming to generate evidence relevant for diagnostic implementation and therapeutic stratification.

## 2 Materials and Methods

### 2.1 Study Population and Clinicopathologic Variables

A retrospective study of patients with GC resected with curative intent at a tertiary hospital in Madrid, Spain, between 2001 and 2011 was conducted. Inclusion criteria comprised histologically confirmed primary gastric adenocarcinoma treated by upfront curative-intent gastrectomy with available formalin-fixed paraffin-embedded tissue. Exclusion criteria included prior neoadjuvant therapy, presence of distant metastatic disease at diagnosis, non-adenocarcinoma histology, or insufficient tumor material for tissue microarray (TMA) construction. A total of 204 cases were finally included, and clinical information was available for 200 patients. Clinical and follow-up data were retrieved from electronic medical records. Tumor recurrence and GC-specific mortality were recorded and used for outcome analyses. Tumor recurrence was defined as radiologic and/or histologically confirmed local, regional, or distant disease relapse after curative surgery. GC-specific mortality was determined based on clinical records indicating death attributable to disease progression, in the absence of an alternative documented cause.

Histopathological features were independently reviewed by two pathologists on routine hematoxylin–eosin–stained whole-tissue sections, according to a standardized morphology recording protocol. In cases of discrepant assessments, slides were jointly reviewed and a consensus diagnosis was reached. Tumors were classified according to Laurén and the World Health Organization (WHO, 2019), and staging was based on the 8th edition of the American Joint Committee on Cancer (AJCC) tumor–node–metastasis (TNM) system. The invasive front pattern was assessed by systematic review of all available histological sections, focusing on the tumor–stromal interface, and tumors were classified according to the predominant growth pattern (expansile vs. infiltrative). Intratumoral inflammatory infiltrates were assessed by initial low-power screening to identify hotspot areas, followed by evaluation at ×20 magnification in one to three representative fields. The use of one to three fields was intended to capture the highest-density areas while accounting for intratumoral variability; when multiple fields were evaluated, the final grade corresponded to the predominant pattern observed across the assessed areas. Inflammatory infiltrates were semi-quantitatively graded as absent/rare, mild–moderate, or marked based on their overall density and visibility within tumor glands and nests, ranging from occasional isolated inflammatory cells to dense infiltrates readily appreciable. The predominant intratumoral inflammatory cell composition was recorded as lymphocyte-predominant or eosinophil/neutrophil-rich only when granulocytic components formed dense infiltrates and/or microabscesses within hotspot areas. Peritumoral inflammatory infiltrates were evaluated in the stroma adjacent to the tumor margin. After low-power screening (×4–×10) to identify areas with the highest inflammatory density, assessment was performed at ×20 magnification and semi-quantitatively graded as absent/low, moderate, or marked based on density, distribution (discontinuous versus continuous), and the presence of inflammatory aggregates dominating the peritumoral stroma.

### 2.2 Immunohistochemical Study

TMA blocks were constructed from formalin-fixed, paraffin-embedded tissue. For each case, two representative regions, corresponding to the tumor center and invasive front, were selected, and 1-mm-diameter cores were obtained using a manual arrayer (MTA-1; Beecher Instruments, Sun Prairie, USA). TMA sections were processed for immunohistochemistry. Immunohistochemical staining for CLDN18.2, p53, and mismatch repair proteins was performed on an automated platform (Dako Omnis; Agilent Technologies, Santa Clara, CA, USA) according to the manufacturer’s instructions. HER2 immunohistochemistry (HercepTest™) was performed using the Dako Autostainer platform (Agilent Technologies).

#### 2.2.1 Claudin 18.2

A rabbit monoclonal anti-CLDN18.2 antibody (clone ZR451; Zytomed Systems GmbH, Berlin, Germany) was used. At our institution, this antibody has undergone internal technical validation for diagnostic use, including comparison with known positive cases stained with the Ventana CLDN18 (43-14A) RxDx Assay (Ventana Medical Systems, Inc., Tucson, AZ, USA) and evaluation on negative control tissues, and is routinely implemented in clinical practice. Appropriate positive and negative controls were included in each staining run.

Staining patterns were recorded as membranous, membranocytoplasmic, or cytoplasmic. Staining intensity was semi-quantitatively categorized as 0 (negative), 1+ (weak), 2+ (moderate), or 3+ (strong). The percentage of positive tumor cells was visually estimated in 10% increments, with intermediate values only recorded when positivity was ≤5%. For each core, a Z-score (intensity × percentage of positive tumor cells) was calculated as a semi-quantitative measure of overall expression. For cutoff-based analyses, only membranous staining (purely membranous or membranocytoplasmic with a clear membranous component) was considered positive, whereas isolated cytoplasmic staining was recorded descriptively but not included for classification purposes.

For clinicopathologic correlations, a single value per case was used, corresponding to the evaluable core or, when both cores were available, to the highest Z-score or the appropriate positivity category based on the selected cutoff (≥10%, ≥50%, or ≥80% of tumor cells with ≥2+ intensity), reflecting the core with the highest level of CLDN18.2 expression rather than an averaged or composite score. Specimens with loss of one core were not classified as missing, as this was considered a TMA-related technical issue without impact on case evaluability.

Given the absence of a universally accepted definition of CLDN18.2 positivity in GC, and the wide variability in staining thresholds applied across published series and meta-analyses [7], multiple pre-specified cutoffs were explored in this study. The ≥80% cutoff corresponds to the current therapeutic definition used in pivotal phase III trials (≥75%, operationalized as ≥80% due to 10% scoring increments) [5,6]. The ≥50% cutoff was selected to reflect intermediate-to-high expression levels consistent with earlier phase II clinical studies, in which eligibility thresholds ranged from 40% to 50% of tumor cells with moderate-to-strong membranous staining [13,14]. The ≥10% threshold was included to capture low-level expression reported in observational series and to evaluate whether clinicopathologic associations were maintained across the full spectrum of CLDN18.2 expression.

In addition, to explore whether very high overall CLDN18.2 expression could identify a biologically distinct subset, “extreme expression” was defined a priori as a Z-score ≥ 250, corresponding to the upper tail of the Z-score distribution within the 0–300 scoring range. This threshold was selected to represent cases with near-maximal combined intensity and extent of staining and was used for exploratory analyses only.

#### 2.2.2 Additional Immunohistochemical Markers

HER2 expression was assessed according to College of American Pathologists guidelines for GC using the HercepTest™ kit (Dako Denmark A/S, Glostrup, Denmark; catalog no. SK001). p53 status was evaluated using a mouse monoclonal anti-p53 antibody (clone DO-7; Dako Denmark A/S, Glostrup, Denmark); overexpression was defined as strong nuclear staining in ≥70% of tumor cells based on established criteria [15,16].

Microsatellite instability status was assessed by immunohistochemistry for MLH1 (clone ES05), PMS2 (clone EP51), MSH2 (clone FE11), and MSH6 (clone EP49) (all from Dako Denmark A/S, Glostrup, Denmark); loss of nuclear expression of at least one marker was interpreted as mismatch repair-deficient (MMRd). Internal non-neoplastic elements were required as positive controls for interpretation, and external controls were run with each staining batch. Isolated loss of PMS2 or MSH6 was classified as MMRd in the same manner as paired losses, and no additional confirmatory molecular testing was performed.

All immunohistochemical evaluations were independently assessed by two pathologists, blinded to clinical and outcome data; discrepant cases were jointly reviewed to reach consensus.

### 2.3 Statistical Analysis

Statistical analyses were performed using IBM SPSS Statistics, version 27.0 (IBM Corp., Armonk, NY, USA). Quantitative variables were summarized as mean, standard deviation (SD), median, interquartile range (IQR), and range; categorical variables as absolute frequencies and percentages. Concordance between cores was evaluated in cases with both valid cores by calculating the overall agreement and Cohen’s κ coefficient. Absolute mean differences in staining percentage, intensity, and Z-score were used as an additional measure of intratumoral heterogeneity. Correlations between continuous variables were assessed using Spearman’s ρ. Positivity rates were calculated using two strategies: any-core (positive if ≥1 core met the cutoff) and both-cores (requiring both cores to meet the cutoff).

Associations between categorical variables were analyzed using Pearson’s *χ*^2^ or likelihood-ratio tests, and continuous variables using the Mann–Whitney U or Kruskal–Wallis tests, or Student’s *t*-test when normality assumptions were met. Survival curves were estimated by the Kaplan–Meier method and compared using the log-rank test. Disease-specific survival was calculated from the date of surgery to death attributable to GC, and recurrence-free survival from the date of surgery to first documented tumor recurrence. Patients without events were censored at last follow-up. A *p*-value < 0.05 was considered statistically significant. No formal adjustment for multiple comparisons was applied; therefore, *p*-values should be interpreted cautiously, particularly for exploratory and subgroup analyses.

### 2.4 Ethical Considerations

The study was approved by the Research Ethics Committee (CEIm) of Hospital Clínico San Carlos (Madrid, Spain), internal code 16/017-E. Owing to its retrospective nature and exclusive use of anonymized data and samples, informed consent was waived. The study was conducted in accordance with the Declaration of Helsinki and current biomedical research regulations.

## 3 Results

### 3.1 Clinical and Pathological Characteristics of the Cohort

A total of 204 cases of GC represented in TMA blocks were included, of which 200 had available clinical and pathological information.

Demographic and morphological features are summarized in Supplementary Table S1. The mean age at diagnosis was 71 years (SD 12), with a median age of 75 years (IQR 66–79), and there was a slight male predominance (54.5%). Most tumors were located in the antrum-pylorus or body of the stomach (89.8%) and corresponded to the intestinal subtype according to Laurén (55.8%). Lymphovascular and perineural invasion were identified in 44.7% and 50.2% of cases, respectively, and tumor necrosis in 26.3%. Most patients were diagnosed at stages III (48.7%) and II (36.8%). During a mean follow-up of 46 months, tumor recurrence occurred in 44.9% of patients, and GC-specific mortality was 29.9%.

All 204 cases were included in the analysis of CLDN18.2 expression and inter-core reproducibility (Section 3.2). Clinicopathologic and prognostic correlations (Section 3.3, Section 3.4, Section 3.5 and Section 3.6) were restricted to the 200 cases with available clinical data.

### 3.2 CLDN18.2 Expression and Inter-Core Reproducibility

A total of 204 GCs were analyzed in TMA format. Among the 408 initial tissue cores, 34 (8.3%) were non-evaluable due to tissue loss or absence of tumor, yielding 374 interpretable cores (technical success rate 91.7%). Most cases (177; 86.8%) had two evaluable cores, whereas 20 (9.8%) had only one, and 7 (3.4%) had no assessable material.

#### 3.2.1 Expression Characteristics

**Figure 1:**
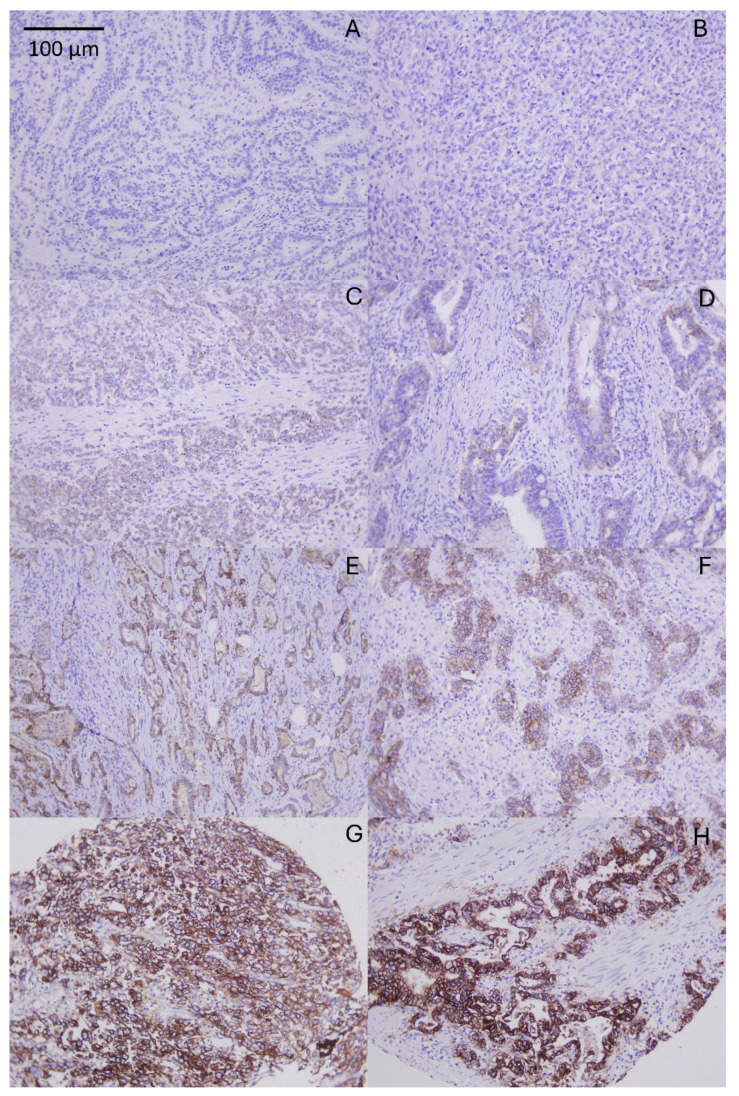
Spectrum of CLDN18.2 immunohistochemical expression in gastric adenocarcinoma; (**A**,**B**) representative cores showing negative membranous staining; (**C**,**D**) representative cores showing mild membranous staining; (**E**,**F**) representative cores showing moderate membranous staining; (**G**,**H**) representative cores showing strong membranous staining. Staining performed with CLDN18.2 antibody clone ZR451 (Zytomed). Original magnification ×100. Scale bar: 100 μm.

Across all evaluable cores, membranous CLDN18.2 staining intensity was 0 in 172 cores (46%), 1+ in 45 (12%), 2+ in 93 (24.9%), and 3+ in 64 (17.1%) (Fig. 1; see Supplementary Fig. S1). The mean percentage of positive tumor cells was 33.9% (SD 41.0), with a median of 5% (IQR 0–80%; range 0–100%). The Z-score (intensity × percentage) showed a mean of 80 (SD 108.5) and a median of 5 (IQR 0–160; range 0–300).

Among positive cores, a purely membranous pattern predominated (180/202, 89.1%; mean Z-score 137.6). Membranocytoplasmic staining occurred mainly in high-expressing cases (mean Z-score 267.9). Pure cytoplasmic staining was rare (1.5%) and weak (mean Z-score 13.3).

#### 3.2.2 Frequency of CLDN18.2 Positivity

**Figure 2:**
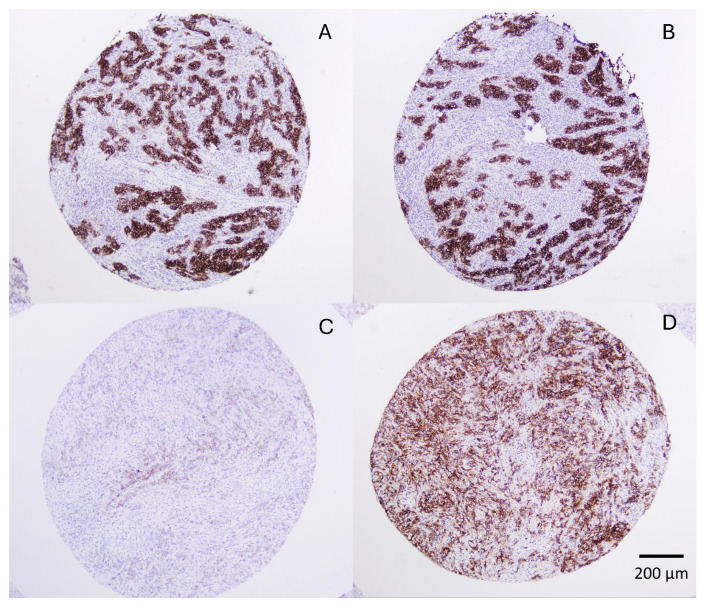
Intratumoral variability of CLDN18.2 expression across duplicate tissue microarray cores; representative cases showing paired TMA cores from two different tumors; (**A**,**B**) concordant case with strong, diffuse membranous staining in both cores; (**C**,**D**) discordant case with core (**C**) showing mild membranous staining in approximately 60% of tumor cells and core (**D**) showing high CLDN18.2 expression (3+, ≥80% of tumor cells). Inter-core reproducibility was high, with an overall agreement of 93.8% and a Cohen’s κ coefficient of 0.871 at the clinical cutoff, defined in this study as ≥80% of tumor cells with ≥2+ intensity (equivalent to the ≥75% threshold used in pivotal clinical trials). Discordant cases accounted for 6.2% of tumors with two evaluable cores. Original magnification ×200. Scale bar: 200 μm.

Using the clinical threshold (≥75% tumor cells with ≥2+ membranous staining, equivalent to ≥80% in our study), 52 of 177 cases with two evaluable cores (29.4%) were positive using the any-core strategy, and 41 (23.2%) were positive when both cores were required to meet criteria. As a result, 11 cases (6.2%) shifted classification when simultaneous positivity in both cores was required (Fig. 2).

**Figure 3:**
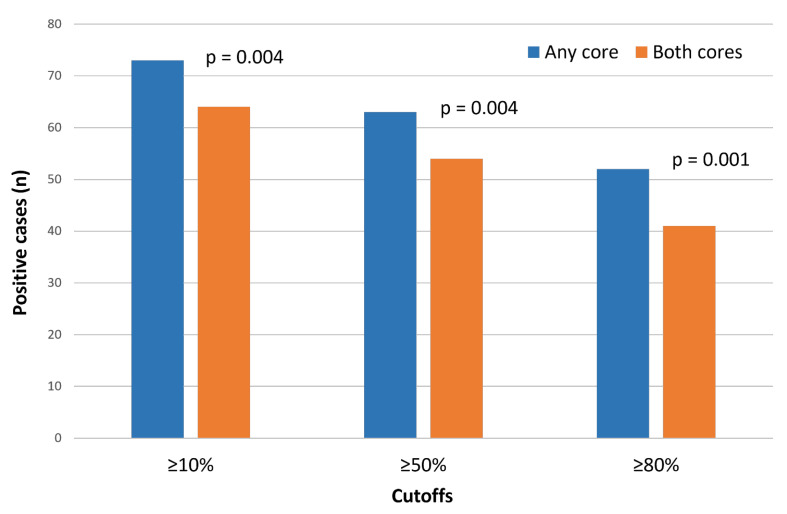
Proportion of CLDN18.2-positive cases according to cutoff and scoring strategy (any-core vs. both-cores) in cases with two evaluable cores (*n* = 177). Differences between scoring strategies were assessed using McNemar’s test in paired samples.

Lowering the cutoff increased the positivity rate to 35.6% (≥50%) and 41.2% (≥10%) using the any-core approach, and to 30.5% (≥50%) and 36.2% (≥10%) using the both-cores method (Fig. 3). The distribution of positive cases according to cutoff and scoring strategy is detailed in Supplementary Table S2.

The any-core strategy consistently yielded higher positivity rates than the both-cores strategy across all cutoffs. These differences were statistically significant at the ≥10%, ≥50%, and ≥80% thresholds (McNemar test: *p* = 0.004, *p* = 0.004, and *p* = 0.001, respectively; Fig. 3 and Supplementary Table S2).

#### 3.2.3 Inter-Core Reproducibility

Inter-core concordance was high. The mean absolute differences between paired cores were 14.7 percentage points for extent of staining (median 10%; range 0–80), 0.37 for intensity (range 0–2), and 26.2 for Z-score (median 15; range 0–210). At the clinical threshold (≥80% with ≥2+), overall agreement was 93.8% with a Cohen’s κ coefficient of 0.871, indicating excellent reproducibility. Agreement remained high at the 50% (90.4%; Cohen’s κ = 0.812) and 10% cutoffs (88.1%; Cohen’s κ = 0.767). Spearman correlations between cores were 0.853 for percentage staining and 0.829 for Z-score.

Discordance was concentrated in marginally expressing tumors (10–70% positive cells and/or moderate intensity without meeting the clinical threshold). Among high-expressing tumors (any-core positive ≥80% with ≥2+; *n* = 52), 11 cases (21.2%) showed one positive and one negative core, representing 6.2% of all paired cases.

### 3.3 Clinical and Histopathological Associations

A total of 200 cases with available clinical information were analyzed. Using the any-core strategy, CLDN18.2 positivity (≥10%, ≥50% or ≥80% of tumor cells with ≥2+ intensity) was observed in 42.4% (81/191), 37.1% (72/194) and 29.9% (58/194) of cases, respectively. CLDN18.2 expression, assessed using these cutoffs, did not show significant associations with most clinicopathological parameters. In particular, no differences were observed with respect to tumor location, size, WHO or Laurén subtype, presence of signet-ring cells or extracellular mucin lakes, tumor grade, lymphovascular or perineural invasion, depth of invasion, lymph node status, or pathological stage.

Across all three cutoffs, CLDN18.2 positivity was consistently associated with sex, invasive front pattern, and tumor necrosis (Table 1). Associations were more pronounced at the ≥50% and ≥80% thresholds, with higher positivity rates in male patients, in tumors with an infiltrative invasive front, and in tumors lacking necrosis (*p* = 0.006–0.043). With the ≥10% cutoff, these differences were less marked and did not consistently reach statistical significance.

CLDN18.2 expression was significantly associated with the intratumoral inflammatory pattern. Tumors positive for CLDN18.2 more frequently showed a mixed inflammatory infiltrate with eosinophils or neutrophils (11–12% vs. 1–3% in negative tumors; *p* = 0.003–0.008). No associations were observed with overall inflammation grade or with peritumoral inflammatory features.

Using the ≥80% cutoff, there was a trend toward a higher frequency of Borrmann type IV tumors among CLDN18.2-positive cases (20% vs. 9.9%; *p* = 0.065) and toward younger age at diagnosis. The trend observed in categorical analysis (proportion of patients ≥75 years, 41.8% vs. 56.7%; *p* = 0.061) became significant when age was analyzed as a continuous variable (mean age 68 vs. 72 years; *p* = 0.049). These associations were not observed with lower cutoffs.

**Table 1:** Clinicopathological variables significantly associated with CLDN18.2 expression.

Variable	Category	≥10%	≥50%	≥80%
Necrosis	**Absent, *n*/N (%)**	**65/138 (47.1)**	**60/140 (42.9)**	**48/140 (34.3)**
	Present, *n*/N (%)	16/51 (31.4)	12/52 (23.1)	10/52 (19.2)
	*χ*^2^ (df), *p*	3.762 (1), 0.052	6.330 (1), 0.012	4.076 (1), 0.043
Invasive front	**Infiltrative, *n*/N (%)**	**58/120 (48.3)**	**54/121 (44.6)**	**43/121 (35.5)**
	Expansive, *n*/N (%)	22/67 (32.8)	17/69 (24.6)	14/69 (20.3)
	*χ*^2^ (df), *p*	4.218 (1), 0.040	7.503 (1), 0.006	4.865 (1), 0.027
Sex	**Male, *n*/N (%)**	**46/100 (46.0)**	**44/102 (43.1)**	**37/102 (36.3**)
	Female, *n*/N (%)	33/89 (37.1)	26/90 (28.9)	20/90 (22.2)
	*χ*^2^ (df), *p*	1.541 (1), 0.215	4.190 (1), 0.041	4.523 (1), 0.033
Type of intratumoral inflammation	**Eosinophil/neutrophil-rich, *n*/N (%)**	**9/10 (90.0)**	**8/10 (80.0)**	**7/10 (70.0)**
	Lymphocyte-predominant, *n*/N (%)	71/169 (42.0)	63/171 (36.8)	51/171 (29.8)
	*χ*^2^ (df), *p*	8.796 (1), 0.003	7.381 (1), 0.007	7.003 (1), 0.008

CLDN18.2 positivity (any-core). Percentages represent the proportion of CLDN18.2-positive tumors within each category. *p*-values were obtained using Pearson’s chi-square test; *χ*^2^ values and degrees of freedom (df) are shown. Bold indicates the category showing the higher proportion of CLDN18.2-positive tumors for each clinicopathological variable.

### 3.4 Biomarker Associations

Among CLDN18.2-positive cases, the proportion of MMR-deficient tumors was 25.9% using the ≥10% cutoff, 20.8% using ≥50%, and 20.7% using ≥80%. CLDN18.2-positive tumors were more frequently MMR-proficient than CLDN18.2-negative tumors, and this difference reached statistical significance at the ≥50% cutoff (*p* = 0.041), with a similar, although non-significant, trend at the ≥80% cutoff (*p* = 0.078).

There was a strong association between CLDN18.2 expression and preserved E-cadherin expression. Complete, intense membranous E-cadherin staining in 100% of tumor cells was present in 87.7% of CLDN18.2-positive tumors using the ≥10% cutoff, 90.3% using ≥50%, and 91.4% using ≥80% (*p* < 0.001 for all thresholds).

No significant differences were observed for HER2 or p53 expression at any of the tested CLDN18.2 thresholds (*p* = 0.23 and *p* = 0.66 at ≥80%, respectively).

Associations between CLDN18.2 expression and biomarker status, using the clinical cutoff (≥80% of tumor cells with ≥2+ intensity), are summarized in Table 2.

**Table 2:** Exploratory associations between CLDN18.2 expression (≥80%) and selected biomarkers.

Biomarker	CLDN18.2+ *n*/N (%)	CLDN18.2− *n*/N (%)	*χ*^2^ (df)	*p*
E-cadherin^1^	53/58 (91.4)	88/132 (66.7)	12.858 (1)	<0.001
MMRd^2^	12/58 (20.7)	45/135 (33.3)	3.116 (1)	0.078
HER2 2+/3+	4/57 (7.0)	4/132 (3.0)	1.561 (1)	0.23
p53 ≥ 70%	9/58 (15.5)	25/136 (18.4)	0.231 (1)	0.66

CLDN18.2 positive = ≥80% of tumor cells with staining intensity ≥2+ (any-core). ^1^Complete and strong E-cadherin expression in 100% of tumor cells; ^2^MMRd = mismatch repair-deficient.

### 3.5 Prognostic Associations

**Figure 4:**
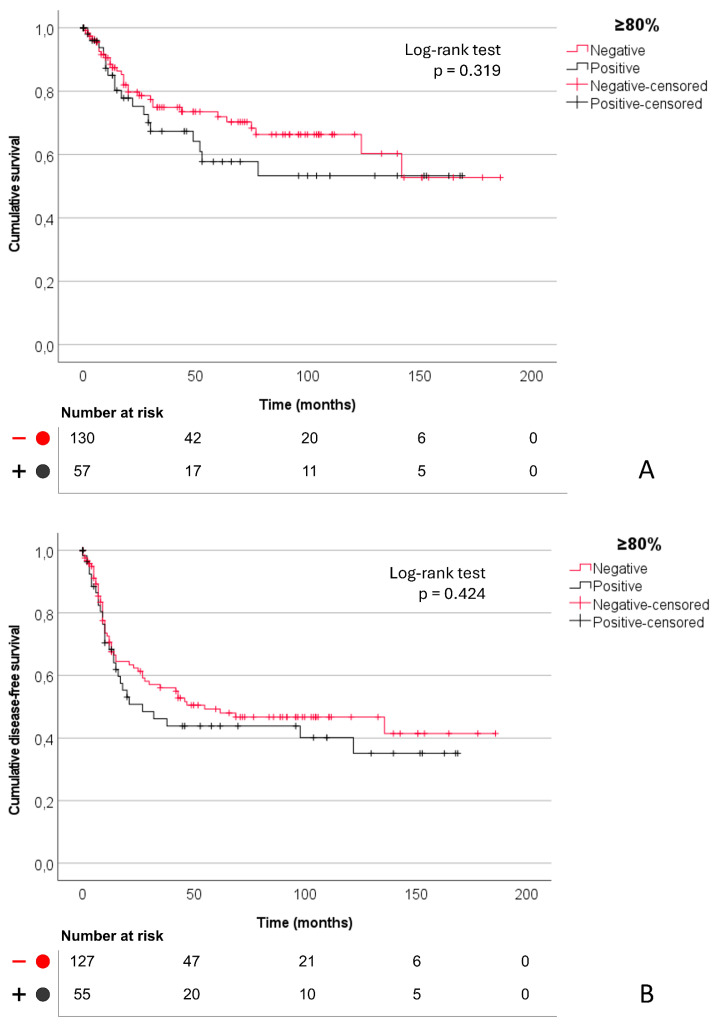
Kaplan–Meier survival curves according to CLDN18.2 expression status (≥80% cutoff): (**A**) disease-specific survival, median survival not reached in either group (log-rank test, *p* = 0.319), and (**B**) recurrence-free survival, with a median survival of 55 months in CLDN18.2-negative tumors and 27 months in CLDN18.2-positive tumors (log-rank test, *p* = 0.424); numbers at risk are shown below each curve.

CLDN18.2 expression was not associated with recurrence or GC-specific mortality (all *p* > 0.05). Kaplan–Meier analysis showed no significant differences in disease-specific survival or recurrence-free survival across cutoffs (log-rank *p* > 0.05). Survival curves corresponding to the clinical cutoff (≥80% of tumor cells with ≥2+ intensity) are shown in Fig. 4.

Analysis of CLDN18.2 expression as a continuous variable (Z-score) using multivariable Cox regression models showed no association with disease-specific survival or recurrence-free survival (Supplementary Table S3).

### 3.6 Extreme CLDN18.2 Expression

Given the absence of prognostic associations using continuous values or standard cutoffs, we evaluated cases with extreme CLDN18.2 expression, defined as a Z-score ≥ 250. This subgroup comprised 28 cases (14.4%).

In univariate analysis, extreme expression was associated with Borrmann type IV appearance (26.0% vs. 10.7%; *p* = 0.029), lymphovascular invasion (67.9% vs. 40.5%; *p* = 0.007), and GC-specific mortality (48.0% vs. 27.2%; *p* = 0.038). A trend toward younger age was observed (≥70 years, 37.0% vs. 54.8%; *p* = 0.087). These results are summarized in Supplementary Table S4. No other significant associations were identified.

**Figure 5:**
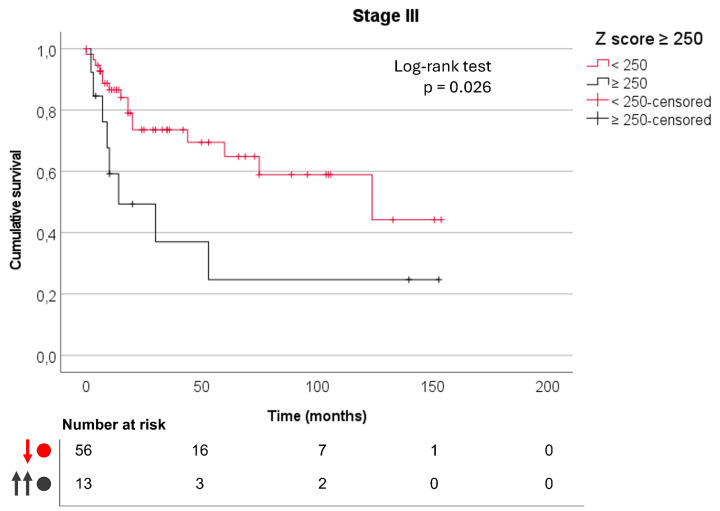
Kaplan–Meier survival curve for disease-specific survival in stage III gastric cancer according to extreme CLDN18.2 expression (Z-score ≥ 250), with a median survival of 124 months in tumors with Z-score < 250 and 14 months in tumors with Z-score ≥ 250 (log-rank test, *p* = 0.026); numbers at risk are shown below the curve.

Kaplan–Meier analysis showed a trend toward worse disease-specific survival (log-rank *p* = 0.089). To assess whether tumor stage could influence this association, a separate analysis was performed restricted to stage III patients (*n* = 90). In this subgroup, extreme CLDN18.2 expression remained associated with GC-specific mortality (61.5% versus 28.6%, *p* = 0.025), and Kaplan–Meier analysis demonstrated significantly worse disease-specific survival (log-rank *p* = 0.026) (Fig. 5). No survival differences were observed in stage III tumors when applying the other CLDN18.2 cutoffs (10%, 50%, and 80%).

## 4 Discussion

CLDN18.2 has both biological and therapeutic relevance in GC. Zolbetuximab, a chimeric monoclonal antibody directed against the extracellular domain of CLDN18.2, selectively binds and induces antibody-dependent and complement-dependent cytotoxicity in tumor cells retaining gastric-type differentiation [17]. Initially, its clinical activity was evaluated in the phase II MONO study, which assessed zolbetuximab as monotherapy in patients with advanced CLDN18.2-positive gastric or gastroesophageal junction adenocarcinoma (≥50% of tumor cells with moderate-to-strong membranous staining) and demonstrated a 9% objective response rate and 23% clinical benefit rate [13]. In the subsequent phase II FAST trial, zolbetuximab combined with EOX chemotherapy (epirubicin, oxaliplatin, and capecitabine) achieved improved survival in patients with advanced CLDN18.2-positive gastric or gastroesophageal junction adenocarcinoma, using a ≥40% cutoff for moderate-to-strong membranous staining [14]. In later pivotal phase III trials, SPOTLIGHT and GLOW [5,6], significant improvements in progression-free and overall survival were confirmed with zolbetuximab plus standard chemotherapy in HER2-negative, CLDN18.2-positive disease (defined as ≥75% of tumor cells with moderate-to-strong staining). These findings led to regulatory approval of zolbetuximab in multiple regions for first-line treatment of CLDN18.2-positive, HER2-negative advanced gastric and gastroesophageal adenocarcinoma.

Moreover, several next-generation CLDN18.2-directed approaches are under active development, including bispecific antibodies (e.g., CLDN18.2/CD3 or CLDN18.2/HER2 constructs), antibody–drug conjugates, and CLDN18.2-targeted CAR-T cells, which have shown encouraging preclinical and early-phase clinical results [18,19,20]. Given this expanding therapeutic landscape, defining the prevalence, reproducibility, and clinicopathologic associations of CLDN18.2 expression in diverse populations is increasingly important.

In addition to its role as a therapeutic biomarker, CLDN18.2 expression also reflects underlying tumor biology and has been investigated in relation to various clinicopathologic and molecular parameters [7]. Nevertheless, interpretation of the available evidence on CLDN18.2 in GC remains challenging due to substantial methodological heterogeneity across studies, including differences in antibody clones, scoring systems, positivity thresholds, and specimen types (biopsies, TMAs, or whole sections), as well as variable definitions of intratumoral heterogeneity [21,22,23,24,25,26]. In addition, most published cohorts are of Asian origin, whereas Western series are comparatively limited and smaller in size [7,8,9,10,11,12,27,28]. These demographic and technical discrepancies likely contribute to variability in reported prevalence, clinicopathologic associations, and prognostic implications.

In this context, our study contributes robust data from a well-characterized Western cohort, using an internally validated antibody routinely employed in diagnostic practice and applying the therapeutic cutoff used in the SPOTLIGHT and GLOW trials [5,6].

### 4.1 Prevalence and Diagnostic Reproducibility

Reported CLDN18.2-positivity rates vary widely (14–88%), largely reflecting methodological differences across studies [12]. Using the clinical threshold (≥75% tumor cells with ≥2+ staining, equivalent to ≥80% in our study), large series report prevalence rates of approximately 30–40% [5,6]. In our cohort, the positivity rate was 29.9%, consistent with these figures, although slightly lower. This may relate to demographic characteristics, including an older median age (75 years), as well as geographic factors.

Regarding intratumoral reproducibility, few studies have evaluated intratumoral heterogeneity of CLDN18.2 in GC, and those available show substantial variation in patient selection, antibodies, sample types and definitions of discordance [21,22,23,24,25,26,29,30]. In whole-section studies, heterogeneity has ranged from 24% to 38.5% [22,23,24]. Other approaches have reported greater discordance with less stringent thresholds but high concordance when the therapeutic cutoff and multiple sampled regions were used [23,25,29]. Some studies have noted greater variability between superficial tumor areas and the invasive front [25].

In our untreated Western cohort, concordance between paired cores from the tumor center and invasive front was excellent (93.8%, κ = 0.871), and discordance was essentially limited to borderline-expressing tumors. Only 6.2% of cases were discordant at the clinical cutoff. These findings support the reliability of CLDN18.2 assessment in limited tissue samples when therapeutic thresholds are applied, provided that adequate endoscopic sampling is ensured, consistent with current diagnostic recommendations for GC [31].

### 4.2 Clinicopathologic Associations

Most studies have not identified consistent clinicopathologic correlates of CLDN18.2 expression [27,32]. Reported associations include sex, although results are conflicting across cohorts [7,11,28], younger age [11,24,33], Borrmann type IV tumors [10,24], tumor location [11], diffuse subtype [11,24,28,34,35], signet-ring cell features [36], and pattern of distant dissemination [11,33], but overall findings have been inconsistent. In our series, CLDN18.2-positive tumors were more frequent in males, demonstrated an infiltrative invasive front, and lacked necrosis. These features suggest a possible association with a viable infiltrative growth pattern, although biological validation is required. We also observed a trend toward younger age and a higher proportion of Borrmann type IV tumors at the ≥80% cutoff, concordant with prior studies [10,24,33], although the effect size was modest.

Regarding the immune microenvironment, few studies have explored the relationship between CLDN18.2 expression and intratumoral inflammatory patterns [8,9,37]. Two reports described increased neutrophilic infiltration in CLDN18.2-positive tumors [8,37]. Similarly, in our cohort, CLDN18.2 expression correlated with a mixed eosinophilic/neutrophilic inflammatory infiltrate, supporting a potential link between claudin expression and immune signaling. This observation may be relevant to future combined strategies integrating targeted agents and immunomodulatory therapies [9].

### 4.3 Immunophenotypic and Biomarker Profile

Available evidence indicates that CLDN18.2 expression is more frequent in genomically stable, HER2-negative tumors, although clear associations with MMR status have not been demonstrated [8,33]. Reports on co-expression with HER2 are likewise conflicting, ranging from inverse correlations to no association [7,8,11,27,33]. Similarly, studies examining PD-L1 expression have yielded mixed results, with both positive and negative associations reported [9,11,23,25].

In our cohort, CLDN18.2 expression was significantly more frequent in MMR-proficient tumors at the ≥50% cutoff, with a similar trend at the clinical threshold. Importantly, CLDN18.2-positive tumors demonstrated a strong association with intense and complete preservation of E-cadherin, reinforcing its relationship with a cohesive epithelial phenotype. No significant associations were observed with HER2 or p53, although interpretation is limited by the low number of HER2-positive cases. Overall, our findings suggest that CLDN18.2 expression in GC is associated with a molecular profile characterized by preserved epithelial cohesion and lack of alternative actionable alterations. This pattern is concordant with the biological features reported in HER2-negative tumors included in the SPOTLIGHT and GLOW trials [5,6], reinforcing the relevance of CLDN18.2 as a therapeutic biomarker within this subgroup.

### 4.4 Prognostic Value

Most studies have not supported a prognostic role for CLDN18.2 expression in GC [10,24,27,32,33,36,38], although some conflicting findings exist [11,39], and recent meta-analyses remain inconclusive, likely due to methodological variation and inconsistent use of clinical thresholds [27,40].

In our cohort, CLDN18.2 expression was not associated with survival when evaluated using standard categorical cutoffs or as a continuous variable. However, tumors with extreme expression (Z-score ≥ 250) exhibited a distinct aggressive profile, characterized by higher rates of Borrmann type IV morphology, increased lymphovascular invasion, and a greater incidence of tumor-related mortality. In addition, disease-specific survival was significantly worse in stage III tumors within this subgroup. Although based on a limited number of cases, these findings suggest that, beyond the binary classification used for therapeutic purposes, markedly elevated CLDN18.2 expression may identify a small subset of biologically aggressive cancers.

From a mechanistic perspective, claudin overexpression may disrupt tight-junction integrity, epithelial polarity, and cytoskeletal dynamics, thereby facilitating invasion and migration [3,4]. Given that CLDN18.2 is physiologically expressed in normal gastric mucosa, some studies have described progressive loss during tumor progression [7] and have associated reduced expression with adverse outcomes [39]. Within this biological context, it is plausible that only expression levels substantially exceeding the physiological range have prognostic significance. Our findings support the existence of a potential “claudin-driven” phenotype in a minority of GCs, which merits further investigation and could have implications for risk stratification and therapeutic decision-making.

### 4.5 Geographic and Population Context

Interpretation of CLDN18.2 prevalence and clinicopathologic associations should account for geographic and population-related factors. As discussed above, most available evidence derives from East Asian cohorts [7,8,9,10,11,12], whereas Western series are fewer and more heterogeneous in design [29,32,33,34,36]. Reported CLDN18.2 positivity varies widely across studies, largely reflecting differences in methodology, positivity thresholds, and cohort composition rather than consistent population-specific patterns [7,27,40]. Asian studies and multiregional trial-screened populations have more frequently reported enrichment of CLDN18.2 expression in diffuse-type tumors [11,28,35] and metastatic patterns with higher peritoneal involvement and lower liver metastasis rates [11,22]. In Western cohorts, metastatic site associations have been described mainly at lower positivity thresholds, with peritoneal metastasis also being more frequent among CLDN18.2-positive tumors [33,36]. Overall, Western series show variable prevalence estimates and less consistent clinicopathologic correlations, underscoring limited cross-study comparability [29,32,33,34,36]. Direct East-West comparisons should therefore be interpreted with caution, as observed patterns may reflect potential underlying biological differences, as well as both study design and cohort composition.

### 4.6 Limitations and Strengths

This study has several limitations. First, its retrospective single-center design may introduce selection bias and limits control over pre-analytical variables. Second, TMA sampling inherently restricts the amount of tumor tissue evaluated and may underestimate intratumoral heterogeneity. However, this was mitigated by the systematic inclusion of two spatially distinct regions (tumor center and invasive front) and the high concordance observed between paired cores. Third, although the cohort size is moderate, subgroup analyses, particularly those focused on extreme CLDN18.2 expression, should be interpreted with caution due to limited case numbers. In addition, given the number of comparisons performed, no formal correction for multiple testing was applied; accordingly, borderline *p*-values were interpreted conservatively. Fourth, the study cohort predates current treatment standards; however, all patients were treatment-naive at the time of tissue acquisition, providing a clear view of intrinsic tumor biology without therapy-related selection effects. Finally, molecular profiling beyond standard immunohistochemistry was not performed, and integration with genomic classifications would provide additional biological insight.

Despite these limitations, the study has important strengths. It represents a large, well-characterized Western series evaluating CLDN18.2 in resected GC, uses an internally validated antibody routinely implemented in diagnostic practice, applies the therapeutic cutoff used in phase III trials, and incorporates rigorous histopathologic review and paired-core sampling to assess intratumoral reproducibility. Importantly, the high concordance observed between tumor center and invasive front supports the reliability of CLDN18.2 assessment in limited tissue samples and aligns with the clinical scenario in which most patients are selected for targeted therapy based on biopsy material.

## 5 Conclusions

In this Western cohort of resected GCs, CLDN18.2 expression showed high analytic and intratumoral reproducibility, with excellent concordance between the tumor center and invasive front. These findings support the reliability of endoscopic biopsies for patient stratification and reinforce the feasibility of CLDN18.2 testing in routine practice. CLDN18.2 positivity was observed in approximately one-third of cases, consistent with pivotal clinical trial data. While expression was largely independent of traditional clinicopathologic variables, positive tumors were more common in males and were associated with an infiltrative invasive front, absence of necrosis, and a mixed eosinophilic/neutrophilic inflammatory pattern, suggesting distinct tumor biology and microenvironment characteristics. Immunophenotypically, CLDN18.2-positive tumors were more frequently MMR-proficient and strongly associated with preserved E-cadherin expression, aligning with a cohesive epithelial phenotype and with molecular features reported in CLDN18.2-selected clinical trial populations.

Standard expression thresholds did not demonstrate prognostic significance; however, cases with extreme CLDN18.2 expression represented a biologically distinct subset characterized by increased lymphovascular invasion, a higher frequency of Borrmann type IV tumors, and higher tumor-related mortality, with disease-specific survival showing a trend in the full cohort and achieving significance in stage III disease. Although based on a limited number of cases, these findings raise the possibility of a potential “claudin-driven” phenotype associated with aggressive behavior and adverse outcomes.

Overall, this study supports the diagnostic robustness and clinical relevance of CLDN18.2 in Western GC. Future studies are needed to better contextualize CLDN18.2 expression patterns, including very high expression levels, and to clarify their significance across the full spectrum of disease.

## 6 Key Points

- *High reliability for clinical use:* CLDN18.2 expression showed excellent intratumoral concordance, supporting accurate evaluation in small biopsy specimens.
- *Prevalence in Western patients:* CLDN18.2 positivity (~30%) mirrors clinical trial populations and supports applicability in Western practice.
- *Distinct pathological profile:* CLDN18.2-positive tumors more commonly showed an infiltrative invasive front, absence of necrosis, and a mixed eosinophilic/neutrophilic infiltrate.
- *Molecular phenotype:* CLDN18.2 positivity was associated with MMR proficiency and preserved E-cadherin, consistent with a cohesive epithelial phenotype.
- *Extreme expression subset:* Very high CLDN18.2 levels identified a small aggressive subgroup with higher lymphovascular invasion and worse survival.

## Data Availability

Data supporting the findings of this study are available from the corresponding author (CDdA) upon reasonable request.

## Supplementary Materials

The supplementary material is available online at https://www.techscience.com/doi/10.32604/or.2026.075609/s1.

## Abbreviations

AJCC TNM	American Joint Committee on Cancer tumor–node–metastasis system
CLDN18.2	Claudin 18.2
GC	Gastric adenocarcinoma
HER2	Human epidermal growth factor receptor 2
IQR	Interquartile range
MMR	Mismatch repair
SD	Standard deviation
TMA	Tissue microarray
WHO	World Health Organization
