# Supplementary material for: Claudin 18.2 Expression in Gastric Adenocarcinoma: Diagnostic Reproducibility and Clinicopathologic Associations in A Western Cohort
Source: Oncol Res. 2026 May 21;34(6):14. doi: 10.32604/or.2026.075609 (PMC13223185; doi:10.32604/or.2026.075609)
Supplement: Supplementary file 1 [file OncolRes-34-75609-s001.zip › TSP_OR_75609-s001.docx]

**Supplementary Table S1.** Main clinicopathological features of the 200 cases with clinical follow-up.

| **Variable** | | **n/N (%)** |
| --- | --- | --- |
| Age, years: mean (SD^a^) ; median (IQR^b^) | | 71 (12); 75 (66–79) |
| Sex (male) | | 108/198 (54.5%) |
| Tumor location | Cardia | 3/176 (1.7%) |
|  | Fundus | 15/176 (8.5%) |
|  | Body | 60/176 (34.1%) |
|  | Antrum-pylorus | 98/176 (55.7%) |
| Macroscopic type | Polypoid | 40/192 (20.8%) |
|  | Flat | 26/192 (13.5%) |
|  | Ulcerated | 57/192 (29.7%) |
|  | Fungating | 69/192 (35.9%) |
| Laurén | Intestinal | 110/197 (55.8%) |
|  | Diffuse | 69/197 (35%) |
|  | Mixed | 18/197 (9.1%) |
| WHO^c^ | Tubular | 111/197 (56.3%) |
|  | Discohesive | 64/197 (32.5%) |
|  | Mucinous | 3/197 (1.5%) |
|  | Mixed | 19/197 (9.6%) |
| Histologic grade (poorly differentiated) | | 106/197 (53.8%) |
| Signet-ring cells | | 83/197 (42.1%) |
| Tumor necrosis | | 52/198 (26.3%) |
| Lymphovascular invasion | | 88/197 (44.7%) |
| Perineural invasion | | 99/197 (50.2%) |
| Invasive front (infiltrative) | | 125/196 (63.8%) |
| Desmoplasia | | 99/194 (51%) |
| Intratumoral inflammatory infiltrate | Absent | 11/195 (5.6%) |
|  | Mild–moderate | 45/195 (23.1%) |
|  | Marked | 139/195 (71.3%) |
| Peritumoral inflammatory infiltrate | | 53/195 (27.2%) |
| pT category | pT1 | 9/195 (4.6%) |
|  | pT2 | 41/195 (21%) |
|  | pT3 | 120/195 (61.5%) |
|  | pT4 | 25/195 (12.8%) |
| pN category | pN0 | 58/187 (31%) |
|  | pN+ | 129/187 (69%) |
| Stage (AJCC^d^) | I | 27/185 (14.6%) |
|  | II | 68/185 (36.8%) |
|  | III | 90/185 (48.7%) |
| Recurrence | | 88/196 (44.9%) |
| Cancer-related death | | 49/189 (29.9%) |
| HER2 2+/3+ | | 8/194 (4.2%) |
| p53 ≥ 70% | | 34/198 (17.2%) |
| MMR^e^-deficient | | 57/199 (28.6%) |

Percentages are calculated using the number of valid cases for each variable (denominators vary due to missing data).

^a^SD: Standard deviation

^b^IQR: Interquartile range

^c^WHO: World Health Organization

^d^AJCC: American Joint Committee on Cancer (8th edition)

^e^MMR: Mismatch repair

**Supplementary Table S2.** CLDN18.2 positivity rates according to cutoff and scoring strategy (any-core vs both-cores), with inter-core discordance.

| **Cutoff** | **Any-core positive n (%)** | **Both-cores positive n (%)** | **Discordant cases (n)** | **McNemar p** |
| --- | --- | --- | --- | --- |
| ≥10% | 73 (41.2%) | 64 (36.2%) | 9 | 0.004 |
| ≥50% | 63 (35.6%) | 54 (30.5%) | 9 | 0.004 |
| ≥80% | 52 (29.4%) | 41 (23.2%) | 11 | 0.001 |

Only cases with two evaluable TMA cores were included (n = 177).

Discordant cases indicate tumors classified as positive by the any-core strategy but negative when both cores were required to meet the cutoff.

**Supplementary Table S3.** Multivariable Cox regression analyses for disease-specific survival and recurrence-free survival including CLDN18.2 Z-score as a continuous variable.

| **Variable** | **DSS^a^ HR^b^ (95% CI^c^)** | **p** | **RFS^d^ HR (95% CI)** | **p** |
| --- | --- | --- | --- | --- |
| CLDN18.2 Z-score (continuous) | 1.001 (0.998–1.003) | 0.566 | 0.999 (0.997–1.001) | 0.526 |
| Laurén type (diffuse vs intestinal) | 2.368 (1.193–4.703) | 0.014 | 1.813 (1.107–2.969) | 0.018 |
| Laurén type (mixed vs intestinal) | 1.340 (0.424–4.233) | 0.618 | 0.982 (0.405–2.379) | 0.967 |
| Lymphovascular invasion (yes vs no) | 1.142 (0.549–2.375) | 0.722 | 1.151 (0.681–1.944) | 0.600 |
| Lymph node ratio (continuous) | 5.195 (1.659–16.272) | 0.005 | 3.517 (1.454–8.503) | 0.005 |
| pT3–4 vs pT1–2 | 1.318 (0.556–3.125) | 0.531 | 1.537 (0.818–2.885) | 0.181 |

Hazard ratios and 95% confidence intervals were obtained from multivariable Cox proportional hazards models including CLDN18.2 Z-score as a continuous variable. Models were adjusted for Laurén classification, lymphovascular invasion, metastatic lymph node ratio, and pT stage (pT3–4 vs pT1–2).

^a^DSS: disease-specific survival

^b^HR: hazard ratio

^c^CI: confidence interval

^d^RFS: recurrence-free survival

**Supplementary Table S4.** Clinicopathological associations with extreme CLDN18.2 expression (Z-score ≥250).

| **Variable** | **Z <250**  **n/N (%)** | **Z ≥250**  **n/N (%)** | **χ²** | **df^a^** | **p** |
| --- | --- | --- | --- | --- | --- |
| Borrmann type IV | 14/131 (10.7%) | 7/27 (26.0%) | 4.77 | 1 | 0.029 |
| Lymphovascular invasion (present) | 66/163 (40.5%) | 19/28 (67.9%) | 7.25 | 1 | 0.007 |
| GC^b^-specific mortality | 37/136 (27.2%) | 12/25 (48.0%) | 4.31 | 1 | 0.038 |
| Age ≥70 years | 86/157 (54.8%) | 10/27 (37.0%) | 2.93 | 1 | 0.087 |

Pearson’s chi-square test was used. Percentages are calculated per column. Only variables showing significant or near-significant associations (p < 0.10) are shown. Denominators vary across variables due to missing data.

^a^df: degrees of freedom

^b^GC: gastric cancer

**
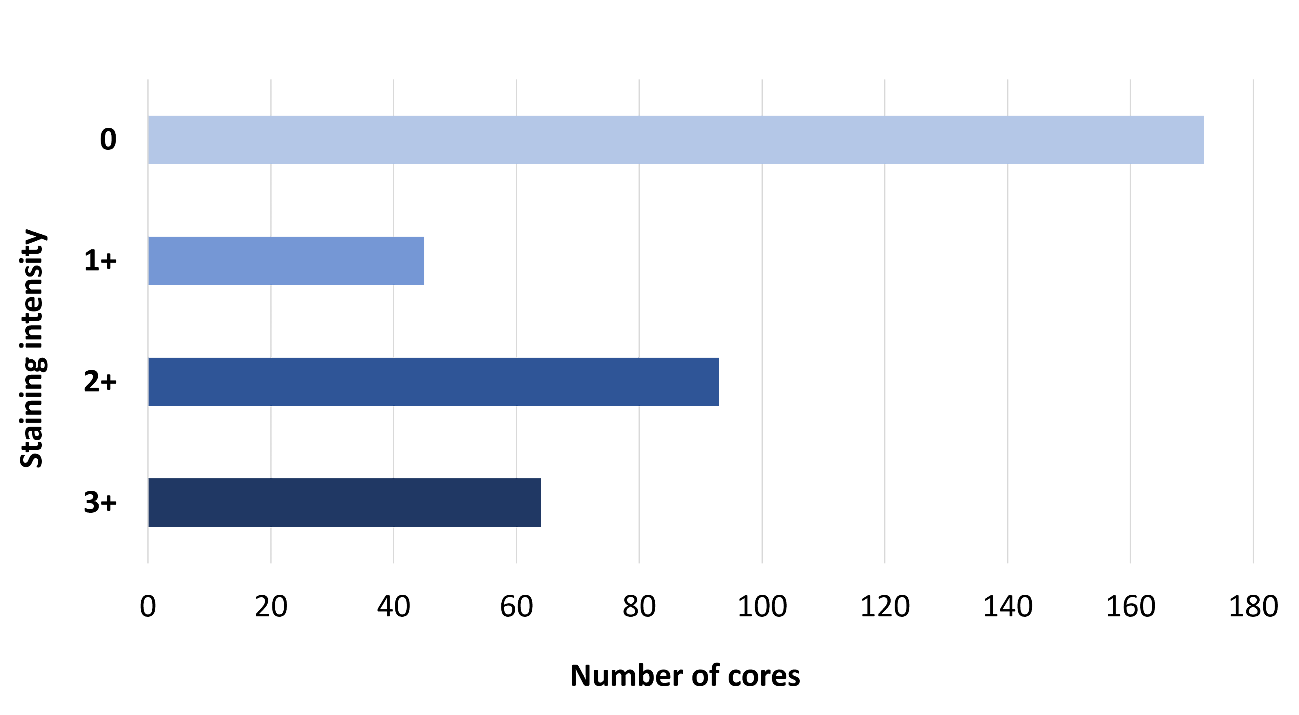
**

**Supplementary Figure S1.** Distribution of CLDN18.2 staining intensity (0, 1+, 2+, 3+) across all evaluable tissue microarray cores. Membranous CLDN18.2 staining intensity was 0 in 172 cores (46%), 1+ in 45 (12%), 2+ in 93 (24.9%), and 3+ in 64 (17.1%).ssss
